# Pneumothorax in Tuberculous Subjects

**Published:** 1905-09-30

**Authors:** 


### PNEUMOTHORAX IN TUBERCULOUS SUBJECTS.

In a lecture on this subject Dr. F. Parkes Weber[1] relates several cases of interest, and then proceeds to deal with the general questions of symptomatology and prognosis. The physical signs of pneumothorax are largely influenced by the presence or absence of pleuritic adhesions. Thus adhesions if situated anteriorly may prevent the displacement of the heart and mediastinum which is so conspicuous a feature of complete pneumothorax. Again, when adhesions limit the extent of the pneumothorax, and more especially in a weakly patient confined to bed, the condition may develop without any of the dyspnœa, pain, and syncope which usually attend its occurrence in a pleural cavity free from the restrictive action of adhesions. Another important point in diagnosis is the frequent absence of one or more of the characteristic physical signs. Thus amphoric breathing may be absent, and at times no breath sounds of any kind can be heard. In some instances the "bell sound" cannot be obtained, in others the affected side appears to move as freely as the opposite chest. Metallic tinkling is by no means always present, yet in some cases it is conspicuous from the outset.

In regard to the course and prognosis, it must be allowed that in many cases pneumothorax is a terminal event, and many patients die almost immediately after its occurrence. More frequently they recover from the immediate symptoms attending the perforation of the pleura, and in the course of a few days fluid, as well as air, is found to be present. This is usually serous at first, and though it may subsequently become purulent, it does not invariably do so. Even when there is much dyspnœa and distress at the onset, these, if the patient survives, usually diminish in the course of a few days, the respiratory mechanism managing to accommodate itself to the altered physical conditions. In a few cases no fluid effusion occurs, and the air undergoes absorption. Probably, in such instances, the condition was caused not directly by a tuberculous focus, but by the rupture of an emphysematous bulla formed below the pleura and in connection with a healed tuberculous lesion. Even when hydropneumothorax follows, the patient sometimes recovers without operative interference.

The ultimate prognosis in tuberculous pneumothorax must depend on the extent of the pulmonary disease, on the general condition of the patient, and on the treatment adopted. Several cases have been recorded of phthisical patients living even for several years and able to enjoy life, after the occurrence of pneumothorax.

In addition to the general hygienic measures demanded in pulmonary tuberculosis pneumothorax demands special consideration in reference both to the immediate and the later treatment. With symptoms of collapse at the onset diffusible stimulants are needed, and such drugs as digitalis and strophanthus may be given. To meet pain and excitement sedatives are required; for example, codeine or small doses of morphine, but care must be exercised, as a decided narcotic effect may be harmful. Aperients to reduce portal congestion may be advisable in very full-blooded patients, and in every case the patient should be disturbed as little as possible. Another measure to meet an emergency is the inhalation of oxygen. Whenever the distress is due to excessive tension ("valvular pneumothorax"), and this probably arises in only a limited number of cases, some of the air may be permitted to escape. To effect this a cannula fitted to an india-rubber tube may be used. The end of the tube may be conducted into a basin containing sterilised water, or the air may be allowed to escape through the cannula into antiseptic dressings. Dr. H. von Schrötter has reported the following treatment in a case of closed right pneumothorax unattended by fluid effusion in a youth of 17 years. Air was aspirated from the pleura and oxygen introduced into the lung through a metal catheter inserted into the right bronchus. The effect of the treatment was watched and controlled by Röntgen-ray examination. The last remains of the pneumothorax were made to disappear by the help of long-continued inspiration of oxygen under increased pressure, using an inhalation mask for the purpose. When hydropneumothorax or pyopneumothorax has developed the question of removal of the fluid arises. If the pulmonary condition is one of advanced disease operative interference beyond mere paracentesis is hardly practicable. But where this is not the case and pus is present in the pleural cavity the case is best treated as an ordinary empyema, though repeated paracentesis sometimes leads to a cure. The same practice when the fluid is serous is also in some cases successful. Many records exist of successful re-section of ribs and drainage of the pleural cavity, the patient surviving the operation and regaining a fair measure of health for several years. Occasionally, but only occasionally, Estländer's operation may be advised. The enormous importance of general hygienic treatment is indicated by the fact that at Davos, out of 20 tuberculous cases recorded by Spengler, in five of the patients (ages between 18 and 30 years) complete apparent cure was obtained. In all serous or sero-purulent effusion was present, and operative treatment was limited to paracentesis. Two of the patients were under observation for several years after their recovery.

[1] Lancet, Sept. 16, 1905.

